# Biopolymers for Biomedical and Pharmaceutical Applications: Recent Advances and Overview of Alginate Electrospinning

**DOI:** 10.3390/nano9030404

**Published:** 2019-03-10

**Authors:** Jolanta Wróblewska-Krepsztul, Tomasz Rydzkowski, Iwona Michalska-Pożoga, Vijay Kumar Thakur

**Affiliations:** 1 Department of Mechanical Engineering, Koszalin University of Technology, Raclawicka 15-17, 75-620 Koszalin, Poland; jolanta.wroblewska-krepsztul@tu.koszalin.pl (J.W.-K.); iwona.michalska-pozoga@tu.koszalin.pl (I.M.-P.); 2 Enhanced Composites and Structures Center, School of Aerospace, Transport and Manufacturing, Cranfield University, Bedfordshire MK43 0AL, UK; 3 Department of Mechanical Engineering, School of Engineering, Shiv Nadar University, Uttar Pradesh 201314, India

**Keywords:** biopolymers, packaging, pharmaceutical, biomedical, electrospinning, alginate

## Abstract

Innovative solutions using biopolymer-based materials made of several constituents seems to be particularly attractive for packaging in biomedical and pharmaceutical applications. In this direction, some progress has been made in extending use of the electrospinning process towards fiber formation based on biopolymers and organic compounds for the preparation of novel packaging materials. Electrospinning can be used to create nanofiber mats characterized by high purity of the material, which can be used to create active and modern biomedical and pharmaceutical packaging. Intelligent medical and biomedical packaging with the use of polymers is a broadly and rapidly growing field of interest for industries and academia. Among various polymers, alginate has found many applications in the food sector, biomedicine, and packaging. For example, in drug delivery systems, a mesh made of nanofibres produced by the electrospinning method is highly desired. Electrospinning for biomedicine is based on the use of biopolymers and natural substances, along with the combination of drugs (such as naproxen, sulfikoxazol) and essential oils with antibacterial properties (such as tocopherol, eugenol). This is a striking method due to the ability of producing nanoscale materials and structures of exceptional quality, allowing the substances to be encapsulated and the drugs/biologically active substances placed on polymer nanofibers. So, in this article we briefly summarize the recent advances on electrospinning of biopolymers with particular emphasis on usage of Alginate for biomedical and pharmaceutical applications.

## 1. Introduction

**Figure 1:**
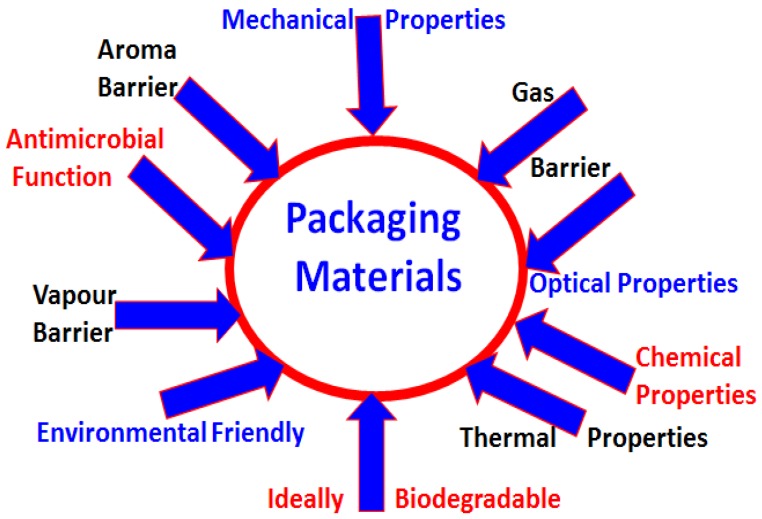
General properties of commonly used packaging materials.

Modern packaging should protect products from external factors, extend the period of maintaining the quality of the product and above all, minimize impact on the environment [1,2,3,4,5,6]. Figure 1 summarizes some of the properties desired in common packaging materials. Detailed properties have been discussed in some excellent review articles, so we won’t be going into more detail [1,2,7,8,9]. In this direction, public awareness is rapidly growing and attracting greater attention to ecology, as well as the use of natural substances for a range of applications from automotive to biomedical [10,11,12,13,14,15,16]. A tendency in the packaging industry to design innovative materials and introduce new solutions is gaining greater attention [17,18]. Active and modern packaging frequently relies on natural substances such as natural polysaccharides [9]. It is imperative to preserve all aspects of environmental safety during production, as well as the effectiveness and safety of use for patients [19,20,21,22,23]. Interestingly, the cyclic olefin copolymer (COC) is most commonly used for tamper-resistant packaging and is a remarkable alternative to a damped polyvinyl chloride (PVC).

**Figure 2:**
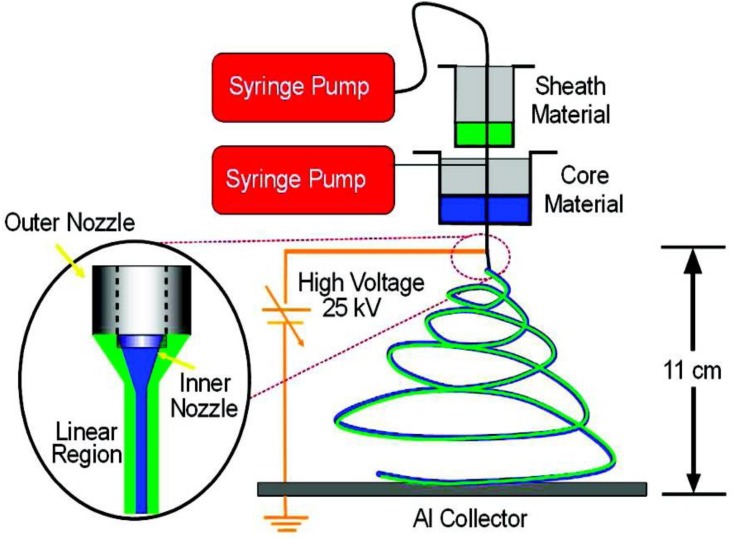
Schematic of coaxial electrospinning setup. The inset shows an illustration of a coaxial jet under applied voltage [39]. Reprinted with permission from Ref. [39]. Copyright American Chemical Society, 2010.

Packaging in medical and biomedical engineering is defined as a technique that enables the closure of a pharmaceutical product from its production to its end use [24]. The role of pharmaceutical packaging is to provide life-saving drugs, surgical devices, nutraceuticals, pills, powders and liquids, to name a few [7,25]. Pharmaceutical packaging influences the isolation and ensures the safety, identity and convenience of using the drug. The packaging must communicate well with the patient so that there are no adverse effects on the health of the patient. The key issue in packaging is also the issue of ecological safety [26,27]. Drug companies that pack drugs are among the industry leaders due to their technological advances. Current trends in industrial research on such new materials are the result of a continuous series of challenges being faced by the industry. The packaging industry is constantly evolving and is an important factor in the development of the pharmaceutical industry and biomedical sciences. Electrospinning of polymers/nanomaterials [28,29] is one of the potential methods in the packaging process that allows for the use of biopolymers [30]/natural substances for the production of medical packaging, dressings, biosensors, medical implants, and is a growing trend in biomedical sciences [10,31,32,33,34,35,36,37,38,39]. Figure 2 illustrates the schematic of a coaxial electrospinning setup. The inset shows an illustration of a coaxial jet under applied voltage [39]. Mercante et al in their excellent review articles have also shown the number of publications that’s involved the use of electrospinning in sensor applications and the number of these publications is increasing day by day [10]. 

Different types of electrospun fibers are being produced for biomedical applications [32]. However, the electrospinning of biopolymers is a very challenging process. For example, in the case of chitosan, its neutralization lead to the loss of chitosan traits by using unsuitable substances. In the production of continuous filaments, suitable solvents should be used so that there is no interruption. In addition, during chitosan nanofiber synthesis, the value of the electric field generated is also crucial. Too high of an electric field causes repulsion between ionic groups of the polymer backbone, which disturbs the formation of continuous filaments. The use of chitin and chitosan nanofibers in biomedical and other applications has been recently reviewed [32,33,34]. Jayakumar et al, in their article, had primarily focused on the properties, preparation and biomedical applications [32]. Electrospinning of silk has also been reported [35]. It is used because of beneficial properties such as non-toxicity and biocompatibility. Due to its positive mechanical properties, it can be used in various temperature and humidity ranges. However, in the case of silk, one of the protein components, sericin, should be removed before biological application because it can cause allergic reactions, for example, in the case of medical dressings [36,37,38].

One of the organisms that is produced as a structural component of silk protein is Bombyx mori [40]. The protein it produces, silk fibroin, is a large crystalline macromolecule consisting of repeating units of amino acids, mainly alanine (A), glycine (G) and serine (S). The fibers that are formed with its participation exhibit good mechanical properties [41]. Researchers have also demonstrated that electrospinning of biopolymer blends of chitin and silk fibroin is possible [42]. In this process, nanofibrous membranes of blended chitosan and silk fibroin were successfully prepared using electrospinning in a HFIP/TFA spinning solvent. With the increment in the content of silk fibroin, the typical diameter of the as-prepared nanofibers was found to increase. The incorporation of silk fibroin was also found to contribute to the enhancement in the mechanical properties of nanofibrous membranes. Furthermore, with the increment in the chitosan content, the antibacterial activity became significantly suitable for wound dressings [42]. Electrospun collagen–chitosan nanofiber having a typical fiber diameter of 434–691 nm was also prepared as a biomimetic extracellular matrix (ECM) for endothelial cells and smooth muscle cells [43]. Different characterization techniques such as FTIR spectra analysis, XRD analysis, DSC and tensile testing were carried out to analyse the developed materials [43]. Double-network (DN) agarose/polyacrylamide nanofibers were prepared by electrospinning [44]. The DN of agarose/polyacrylamide (PAAm) nano fibers developed using simultaneous photo-polymerization and electrospinning. Different characterization techniques were used to confirm the realization of a crosslinked double-network. In comparison to the pristine agarose, the electrospun fibrous agarose/PAAm demonstrated 66.66% enhancement in the strength [44]. Similarly, electrospinning of alginate is being carried out for a number of applications [45]. Alginate is an important biopolymer with vast potential [46]. 

### 1.1. Synthetic Polymers 

Different types of polymers ranging from natural to synthetic are rapidly becoming the most interesting subject of research in the sector of the biomedical industry [47]. They are often used in the packaging of medicines [48,49], as well as in the development of flexible ampoule/syringes that are more easy to use. However, adsorption and migration of the bioactive substance to the polymer changes in pH, permeability of oxygen, optical properties and the release of leached components affect their use and should be taken into account [50,51]. Interaction of the different outer components not only affects the drug but also the function of the polymeric container. Polyolefins, high-density polyethylene (HDPE) or polypropylene (PP) are some of the most common polymers used for the production of vials. Often, multilayer containers are developed to achieve such requirements as inertia, oxygen or UV protection. Polycyclic and olefinic polymers and copolymers (Daikyo Crystal Zeniths) have been used for filling polymer syringes [51,52]. Devices such as PVC tubes containing di-2- ethylhexylphthalate (DEHP) plasticizer are used in dialysis for blood supply or extracorporeal oxygenation. The bags containing the polymer are used to donate blood and store blood products. Due to lipophilicity, the plasticizer is transferred from the polymer surface to lipids and red blood cell membranes [53]. It has been found that the plasticizer in blood bags reduces haemolysis of red blood cells by about 50% compared to blood stored in non-plastic containers, which improves the quality of the blood product [50]. Tubes for extracorporeal circulation are often heparinized to reduce the coagulation, which causes intense contact with PVC and increases thrombogenicity [54]. 

For the storage of red blood cells, an alternative plasticizer such as butyryl-trihexyl-citrate (BTHC) or di-iso-nonyl-1, 2-cyclohexanedicarboxylate (DINCH) is used. Polyolefins as alternative polymers are used to store platelets [55,56]. Polyethylene and polyurethanes are used to create tubes. Tubes of positronic pumps are usually made of silicone [51]. Hemodialysis membranes are produced as bundles of hollow fibers with a surface in contact with blood. The technical requirements concern mainly the permeability for substances smaller than albumin, preventing the passage of impurities from the dialysate to the blood, and the compatibility of the membrane with blood. Previously, dialysis membranes were made of cellulose [51,57]. The hydroxyl groups were replaced with acetylene derivatives or other modified additives, preventing the activation of the complement system and the associated leukocyte activation and leukocyte sequestration in the lungs [51,57]. Synthetic membranes consist of a hydrophobic base material and hydrophilic components. The polyaryl sulfone co-precipitation membranes, polysulfone (PSf) and polyvinylprolidone (PVP) membranes are the most popular for a number of applications [58]. In addition, other membrane materials such as polyamide (PA), polycarbonate (PC) and polyacrylonitrile (PAN), PMMA, polyester polymer alloy (PEPA), ethylene vinyl alcohol copolymer (EVAL), and molecular thin nanoporous silicon diaphragms are also used. Poly (ethylene glycol) (PEG) is used in membranes to improve compatibility with blood [59]. Polymer stents used in the upper sections of the ureter are designed to overcome the problems of sperm infection. Silicone is the best biocompatible material with the lowest incrustation tendency. Its use is limited by low mechanical stiffness and high resistance. Therefore, polyurethane products with better mechanical properties than silicone were optimized [51,60]. The stents were coated with glycosaminoglycans (GAGs, heparin or pentosan polysulfone), phosphorylcholine, which increases the comfort of patients, reduces bacterial colonization and encrustation [51,60]. 

### 1.2. Biopolymers: Structure of Alginate

Recently, there has been a great thrust on the usage of biopolymers for a number of applications, especially in the biomedical and pharmaceutical [61,62,63,64,65]. The functional efficiency of the biopolymer molecules depends on the composition, physicochemical properties and structural features [66,67,68]. It is possible to rationally design the composition and structure of the biopolymer to obtain the appropriate functional attributes [23]. The internal structure of the polymer molecule determines many functional characteristics such as permeability, chargeability and integrity [69]. The stability of the biopolymer particles and their ability to aggregate is influenced by the electrical characteristics. Biopolymer particles with a high electric charge will repel and prevent aggregation. Molecules of biopolymers and their electrical properties influence the interaction with other molecules present in the surrounding environment [69]. Among natural biopolymers, alginate is one of the most popular and intensely studied [70,71]. It is an anionic biopolymer consisting of units of mannuronic acid and guluronic acid in irregular blocks [72]. Mannuronic acid and guluronic acid are linked by glycosidic linkages [73,74,75]. Mannuronic acid forms β (1 → 4) bonds and α bonds (1 → 4) with guluronic acid [76]. The stiffness of molecular chains is ensured by the rigid and bent conformations of guluronic acid [77,78]. Hadas and Simcha have recently reported their interesting work on the characterization of sodium alginate and calcium alginate with particular emphasis on their structure [79]. Different properties and applications of alginate have also been reviewed [80]. The properties of alginates used in biomedicine can be shaped by modifying the availability of their hydroxyl and carboxyl groups [81]. It affects the properties of alginates, such as solubility, hydrophobicity and their biological activity. Alginate hydrogels were created by crosslinking polymer chains [82]. The chemical properties of alginate hydrogels were found to depend on the cross-linking density of the chain [83]. One of the methods used in the design of alginate hydrogels is intermolecular cross-linking, in which only the alginate guluron groups react with the divalent cation most often the calcium used to gel the alginate [84]. Marguerite has summarized the applications of alginate especially for packaging in an excellent review article, so we won’t go into detail [85]. 

## 2. Electrospun Antioxidant/Antibacterial Materials for Medical Packaging 

**Figure 3:**
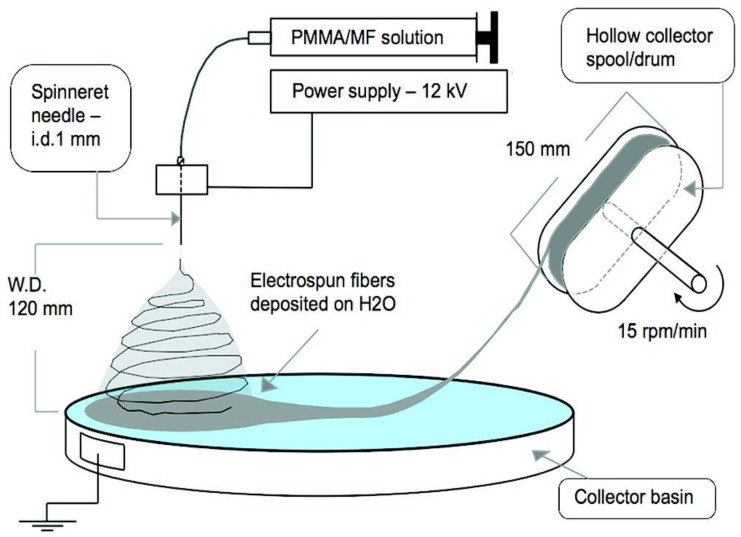
Electrospinning setup with a spinneret–water surface working distance of 120 mm and a spool running at 15 rpm/min. The spool (hollow) was designed to collect fibers with minimal spool–fiber yarn contact [94]. Reprinted with permission from Ref [94]. Copyright American Chemical Society, 2010.

Electrospinning is one of the most prominent techniques currently being used in the development of a number of products for biomedical applications, as discussed in the preceding section [86,87,88]. The electrospun creates ultrathin fibers collected in a random pattern. The mats produced in this way are used as filters, catalytic carriers, dressings and drug delivery systems [89]. Currently, scientific research focuses on the use of nanofiber properties and the focus on determining the parameters of electrospinning of biopolymers for medical and pharmaceutical applications [90,91,92]. In this technique, varying electrified fields are applied to produce polymer filaments, which can then be embedded in various device platforms (briefed in introduction section). The basic device for electrospinning consists of four parts: a syringe containing a polymer solution, a metallic needle, a power source and a metal collector (with variable construction) [93]. The polymer solution is extruded through the tip of the needle by forming a polymer stream that is leached out of the needle and expands as a result of deflection and dissipation of the electric charge voltage of the solution surface. The basic set for electrospun fibers is shown in Figure 3 [94]. Electrospinning of anisotropic fiber yarns (Biohybrid) was carried out via the extraction of microfibrils from bacterial cellulose networks [94]. In the equipment, a plastic syringe of 5 mL was used as the polymer reservoir in the continuous feed system. An aluminum reservoir that was filled with water was used and the fibers were subsequently collected on the surface of the water 120 mm from the needle tippet [94]. 

The polymeric solution stream flows towards the metal manifold with simultaneous evaporation of the solvent and then the nanofibers are deposited on the collector surface. When nanoparticles are deposited instead of nanofibers, the process takes place using electrospray [95,96]. Electrospinning is a fiber production process with a diameter of 0.01 to 10 μm by using electrostatic forces. In the electrospinning technique, a syringe filled with a polymer solution has a high potential source from 10 to 30 kV. The electrical voltage produces free electrons, ions or pairs of ions attracted to the electric field [97]. One of the elements is Taylor’s cone, in which the polarity of the solution depends on the generator’s voltage. The drive between similar charges in the electric space acts against the surface tension and fluid elasticity in the polymer solution, distorting the drop to the shape of the cone structure. In addition to the critical charge density, the Taylor taper becomes unstable and the liquid stream is released from the tip of the cone. Then, in the presence of the electric field, the polymer stream is formed, creating a light continuous form of the fiber. The fluid stream through continuous stretching becomes unstable because the number of spiral paths accumulated on the collector electrode increases [98]. This area is called whipping region. Polarized electrospun nanofibers move down to collision with less collector plate potential. The collector’s morphology affects the straightening of the fiber. Different collectors are used, such as drum rotary collector, movable belt collector and straight mesh collector. To produce nanofibers with controlled morphology, parameters such as molecular weight of the polymer, surface tension, solution viscosity, solution conductivity, flow rate, temperature, collector morphology and distance of the collector apex are optimized [99]. It has been found that the diameter of the fibers increases with increasing concentration and viscosity. Increasing the molecular weight reduces the risk of interference on fibers such as balls and drops. It was determined that nanofibers can be formed with a variety of secondary structures. Thus, nanofibers with a core-shell structure, with an empty interior or with a porous structure may be formed [99,100]. As a drug delivery system, a mesh made of nanofibres produced by the electrospinning method offers a number of advantages. This is an attractive method due to its ability to produce nanoscale materials and structures of exceptional quality [101]. This allows substances to be encapsulated and drugs and biologically active substances placed on polymer nanofibers [102]. Biologically active substances can be attached to nanofibers like a mesh in the electrospinning process. In this way, antibacterial and antioxidant materials are created for medical applications [103,104]. Coating materials containing chitosan formulations with antioxidant and antifungal properties were also formed [88,105].

By choosing a base mesh polymer, it can be easily designed to have improved mechanical properties, biocompatibility and cellular response, which makes mesh a good medical product in the nanocomposite materials sector. The electrospinning creates a macroporous scaffolding containing randomly oriented or aligned nanofibres on which the drug is placed. Electrospun nanofiber scaffolds provide the optimal environment for vaccinated cells [88]. Therapeutic compounds such as lipophilic and hydrophilic drugs, proteins, antimicrobials, etc. can be incorporated into the polymer nanofiber mesh by using monoaxial or coaxial electrospinning [106]. 

Nanofibers can help treat skin damage and can be considered a substitute for skin tissue [107]. The drug enclosed in the nanofiber mesh is released by various mechanisms when the nanofibrils mesh is swollen, biodegradable or absorbed by the human body. As an effective dressing, it inhibits bacterial growth during wound healing. The lower drug release rate allows for non-wound healing of wounds from a few days to several weeks [100,105]. Polymer after electrospinning acts as a barrier controlling the release of loaded molecules. The advantage of this method is the ability to close almost all drugs (especially hydrophobic) in the core, regardless of the drug-polymer interaction. Drugs, proteins, uptake factors and genes can be included in nanofibers [105]. 

Silver nanoparticles exhibit the capability to interact with bacteria. This allows the size of silver nanoparticles 1–10 nm in diameter. The smaller size provides better antibacterial activity. Scientific research has proven the antibacterial activity of electrospun nanofibers containing polylactic acid and silver nanoparticles (AgNPs) against Staphylococcus aureus and *Escherichia coli*. Fibers of silver nanoparticles and polyethylene oxide were mixed with polyurethane fibers exhibiting antibacterial activity on *Escherichia coli* [103,105]. Antimicrobial efficacy increases with an increase in the concentration of silver. The activity can also be increased by reducing silver nanoparticles. Smaller silver nanoparticles have the ability to disperse the bacterial membrane and inhibit bacterial growth. Antimicrobial activity of electrospun nanofibers results from the distribution of silver nanoparticles to electrospun nanofibers. Increased access of silver nanoparticles to electrospun nanofibers increases microbial capacity [108,109,110]. Polyacrylonitrile fibers combined with silver nanoparticles showed antibacterial activity on gram positive Bacillus cereus and gram negative *Escherichia coli*. The fibers formed from the combination of silver nanoparticles and Nylon 6 have an antibacterial effect on the gram of negative *Escherichia coli* and gram positive on Staphylococcus aureus [111]. 

Antioxidative effects of electrospun nanofibers have also been proven by obtaining multifunctional biomaterials, especially through the use of vitamin E and natural biopolymers [105]. Fibers resulting from the combination of polylactic acid, silver nanoparticles and vit. E inhibited the growth of *Escherichia coli* up to 100%. The release time of silver ions from nanofibres immersed in water lasted up to 10 days. It was proved that the combination of polylactic acid nanofibres, silver nanoparticles and vitamin E showed antioxidant activity in studies on fresh apple juice [112]. 

It has been proven that nanofibers act as a membrane that actively reduces polyphenol oxidase activity. Such materials can be used for preservation in the food industry in fruit and juice packaging [105,112]. Nanofibers can be coated with biocompatible polymers (hydrolysed collagen, elastin, hyaluronic acid, chondroitin sulfate). In this way, the electrospun fibers are coated with pure polyurethane to use the substance to improve the antibacterial effect of urinary catheters. The membranes thus obtained, have good antimicrobial activity on *Escherichia coli*, Salmonella typhimurium, Listeria monocytogenes [105]. 

Much research has been focused on the learning and operation of plant extracts using electrospun fibers [113,114]. Researchers have managed to encapsulate with the use of electrospun fibers several raw plant extracts such as Centella asiatica, baicalein, green tea, Garcinia mangostena, Tecomella undulata, aloe vera, Grewia mollis, chamomille, grape seed, Calendula officinalis, Indigofera aspalathoides, Azadirachta indica, Memecylon edule and Myristica andamanica [112]. Also, essential oils such as linalool, pinene, eugenol and cymene are used [115,116,117]. Antibacterial action with essential oils is determined by their hydrophobic nature. Bioactivity of essential oils combined using electrospun fibers to create scaffolding of fibers with antibacterial properties and thermo-mechanically controlled mobility. Electrospinning of chitosan nanoparticles and cinnamon essential oils in the ratio 1:1 has been demonstrated [112]. Fibers with a diameter of 38-55 nm were formed. The fibers were prepared from an aqueous solution containing 5% w/v acetic acid and various concentrations of essential oil (0.5 and 5.0% by volume). After fabrication, the fibers were cross-linked with glutaraldehyde to increase the stability of their chemical properties. The activity of chitosan nanofibres and ether oil particles has been proven in action against *P. aeruginosa*, *E. coli*. Efficacy of the essential oil was demonstrated in a study in which cellulose acetate was used as a polymer matrix [118]. Fibers containing essential oils of peppermint and lemongrass were also analyzed. The generated scaffolds inhibited *E. coli* proliferation and were non-toxic to fibroblasts and keratinocytes [116]. The Shikonin component of Lithospermum erytrorhizon root dried was also used in the electrospinning technique [116]. It has anticancer, antioxidant, anti-inflammatory and antibacterial effects. Shikonina was encapsulated and placed on PCL/poly (trimethylene carbonate) fibers. Note that the drug was released in the first hour at an increased rate and then for 48 h at a fixed dose. The shikonin-laden fibers showed a profound effect against *E. coli and S. aureus*. The healing properties were also tested in combination with alkannins, which are naturally occurring hydroxynaphthoquinone. The connection was used for the preparation of topical and transdermal patches. Cellulose acetate, poly (lactic acid) (PLA) and two different poly (lactic-glycol) mixtures were used as a matrix. Inclusion complexes of cyclodextrins (CD-IC) with plant extracts and complexes with eugenol (EG) were also formed [119]. Eugenol has a bactericidal effect and, in combination with inclusion cyclodextrins, has been used in electrospun fibers. The use of cyclodextrins complexes, increases the solubility of natural extracts in water affects antioxidant and antibacterial activity. Chitosan-based dressings were also developed for biomedical applications. A mixture of chitosan, TiO_2_, poly (*N*-vinyl pyrrolidone) a synthetic polymer with good biocompatibility was prepared [120]. The membrane thus formed showed high activity against the microorganisms *S. aureus, B. subtilis, E. coli, P. aeruginosa*. The use of a membrane of chitosan, poly (*N*-vinylpyrrolidone) and silver oxide showed a similar effect, but with the added advantage of film transparency, allowing for observation of the wound [120].

**Figure 4:**
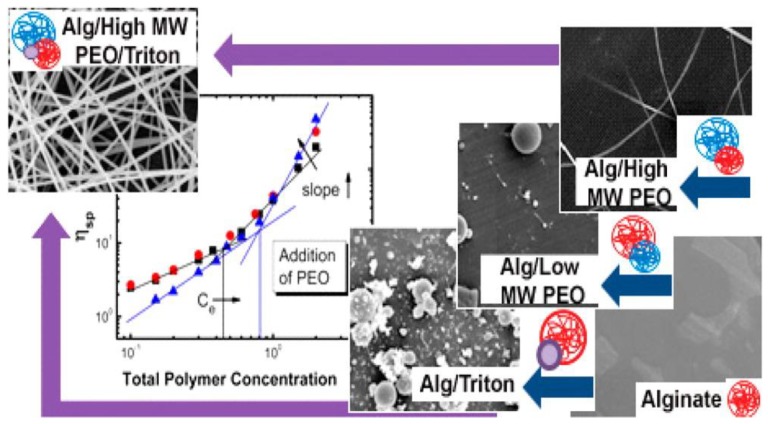
Schematic for the synthesis of alginate–polyethylene oxide blend nanofibers and the role of the carrier polymer in electrospinning. Reprinted with permission from Ref [121]. Copyright American Chemical Society, 2013.

Sodium alginate based nanofibers were also synthesized using polyethylene oxide (PEO) as “carrier polymer” [121]. It was concluded from the study that the sodium alginate on its own could not be electrospun. However, with the addition of a suitable polymer, it can be easily electrospun [121] and the PEO–PEO interactions with high molecular-weight entangled PEO were the key to “carrying” the alginate from the prepared solution to synthesise the fibers using electrospinning (Figure 4).

**Figure 5:**
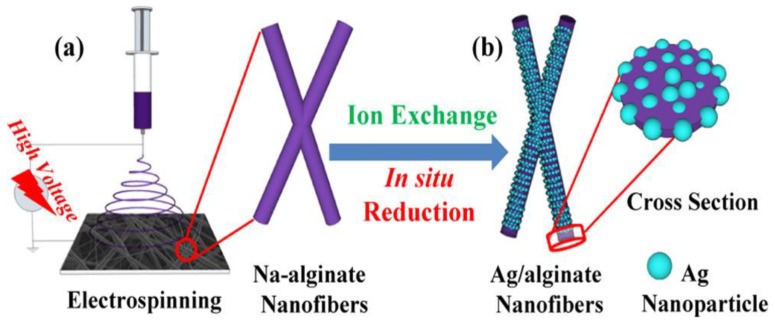
Schematic diagram illustrating the fabrication process of Ag/alginate nanofibers. (**a**) Na-alginate nanofibers prepared by electrospinning. (**b**) Ion-exchange and in situ reduction processes. Reprinted with permission from Ref. [124]. Copyright American Chemical Society, 2018.

**Figure 6:**
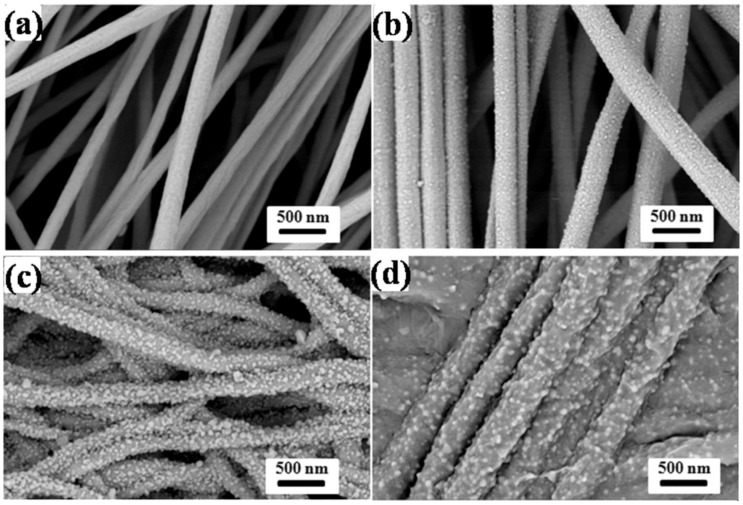
SEM images of (**a**) as-electrospun Na-alginate nanofibers and (**b**–**d**) Ag/alginate nanofibers obtained at different reduction times: (**b**) 10, (**c**) 20, and (**d**) 30 min. Reprinted with permission from Ref. [124]. Copyright American Chemical Society, 2018.

In other studies, it was proved that nanofiber mats containing silver ions were more active compared to nanofibre mats without silver nanoparticles [122,123]. To monitor the human breath, smart fabrics were synthesized via electrospinning of the in situ assembly of well-dispersed Ag nanoparticles [124]. In this work, lightweight and flexible Ag/alginate nanofiber sensor were successfully developed that have the capability to sensitively monitor human breath (Figure 5 and Figure 6). Figure 5 shows the schematic for the fabrication of Ag/alginate nanofibers and Figure 6 shows the SEM images of different alginate nanofibers. 

Other synthetic polymers such as polyurethane, polyacrylonitrile, poly (acrylamide), poly (nitrilesulfonic acid sodium salt, poly (sulphobetaine methacrylate) were also used to develop wound dressings. Fluoroquinolones and norfloxacin were attached to polyphosphates by chemical modification using amino acid esters (alanine, glycine, and phenylalanine) as chain extenders, and these components were then used to create nanofibers by electrospinning [125].

Antimicrobial biodegradable multilayer systems were developed using electrospinning, especially the active multilayer structures based on natural polymers [126]. The system was developed in such a way that it consisted of different layers; for example, alginate-based film as outer layer; using a PHBV8 film as outer layer or no outer layer. Different characterizations such as oxygen and water vapour permeabilities, intermolecular arrangement, transparency and thermal properties were evaluated. In addition the antimicrobial activity was also evaluated. Alginate based coatings for Food Packaging Applications have been recently reviewed by Tugce et al. [127]. In this article, authors have summarized the recent advances on the usage of alginate for recent edible coatings.

## 3. Biopolymers for Biomedical and Pharmaceutical Packaging 

**Figure 7:**
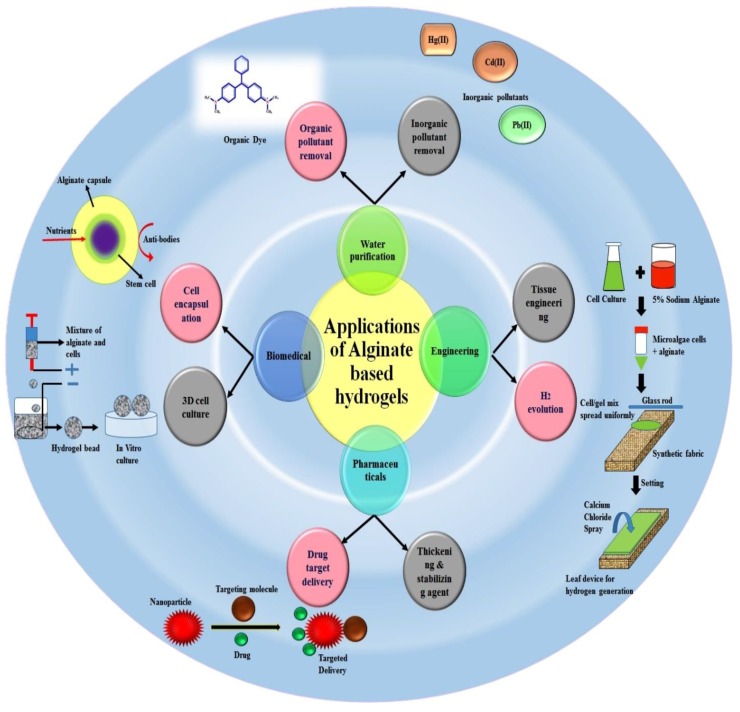
Various applied applications of alginate based hydrogels [135]. Reprinted with permission from Ref. [135]. Copyright Elsevier, 2018.

Active and modern packaging biomaterials contain natural substances that are abundantly found in nature [1,11,128]. Biomaterials are often based on natural polysaccharides [129,130,131,132,133]. Among polysaccharides, Alginates have found applications in the food sector, water purification, biomedicine and packaging [73,134,135,136,137,138,139]. Algae contain nutrients such as vitamins, salts, iodine and sterols. Organisms containing large amounts of alginate in the cell walls are the brown algae Phaeophyceae such as Fucus, Laminaria, and Aseophyllum. The amount of alginates obtained generally depends on the species of algae and the extraction methods used [140]. They are linear polymers composed of (1→4)-α-L-guluronic acid blocks (GG) β-D-mannuronic acid blocks (MM) additionally, of heteropolymeric sequences of M and G (MG blocks) [74,80]. In biomedicine, alginates are used for controlled drug release, encapsulation, scaffolds in ligaments, tissue engineering and in dentistry for the preparation of forms in the presence of slow-release calcium salt [141]. The pharmaceutical industry uses purified alginates for dispersion or stabilization of substances. In biomedicine, alginates are used for controlled drug release, encapsulation, scaffolds in ligaments, tissue engineering and in dentistry for the preparation of forms in the presence of slow-release calcium salt. Figure 7 shows the application areas of alginate hydrogels. The alginate produces edible coatings with good barrier and mechanical properties allowing the protection of active ingredients by encapsulation [3]. Garlic oil is often added as a natural antibacterial agent in such coatings. Alginate is partially dusted with calcium and mixed with starch to obtain high water retention in the paper coating. This is important in order to obtain a uniform mass and coating by pressing to improve its rheology [85]. Alginates have found a number of applications in biomedical sciences as wound dressing materials [142]. Especially sodium alginate used in the form of a hydrogel has stimulated more and more scientific interest due to its physicochemical properties. Materials made of alginate are considered to be friendly to humans due to tissue biocompatibility, which allows for their use in biomedical engineering [135,140]. Highly absorbent dressing materials are formed by the production of wet spinning fibers. With the addition of calcium and sodium, high-absorbency sodium and calcium fibers were produced. Antimicrobial fibers were also formed by adding alginic acid or silver. By adding zinc, the fibers that generate the immune system were created. Fibers for immobilizing or supporting bioactive molecules were readily prepared [143]. Antimicrobial properties were imparted to cotton fabrics employing alginate–quaternary ammonium complex nanoparticles [144]. Using the ionic gelation method, a new type of nanoparticle (average size of 99 nm) that was composed of sodium alginate (SA) and 3-(trimethoxysilyl) propyl-octadecyldimethylammonium chloride (TSA) was synthesized. Fibers exhibited an efficient antimicrobial activity that was even maintained after 30 laundry cycles (non-leaching antimicrobial agent) [144]. Ionic gelation was used to develop a new class of nanoparticles that consists of sodium alginate (SA) and 3-(trimethoxysilyl) propyl-octadecyldimethylammonium chloride (TSA). The ratio of SA/TSA was found to exhibit a significant effect on the average size of the SA–TSA nanoparticles. Nanoparticles having an average size of 99 nm were selected for the study and, after using a pad-dry-cure method, were loaded onto cotton fabrics. Different characterization techniques were used to analyse the treated fabrics. It was concluded from the study that the SA–TSA nanoparticles exhibit high potential to be used as a non-leaching agent imparting robust antimicrobial characteristics to the studied cotton fabrics [144]. The new generation of medical textiles marks the field of expansion for scientists and researchers [145]. The current dressings are non-toxic, bacteriostatic, antiviral, non-allergic, hemostatic, highly absorbent and, above all, biocompatible. It is possible to modify them so that they contain medicines with some mechanical properties. Current textile materials in modern packaging are also highly diverse. These include tapes, fabrics, non-woven fabrics, knitted fabrics, composite materials [16,146]. Lignin and cellulose [147,148] are the most abundant natural polymers available as by-products of various industries. Recent scientific reports show that lignin is increasingly used as an essential component of hydrogels [149]. This allows for the creation of various types of materials, especially in the medical or pharmaceutical sector [150]. It is also possible to add additives to dressings such as odor absorbing, soothing pain and irritation. 

Nanofibres are being used in the treatment of wounds due to interesting properties such as fiber diameter at the nanoscale, porosity, low weight. Polymers from various natural resources are attracting the attention of more and more scientists for exploring their use in wide range of application [151,152,153]. 

**Figure 8:**
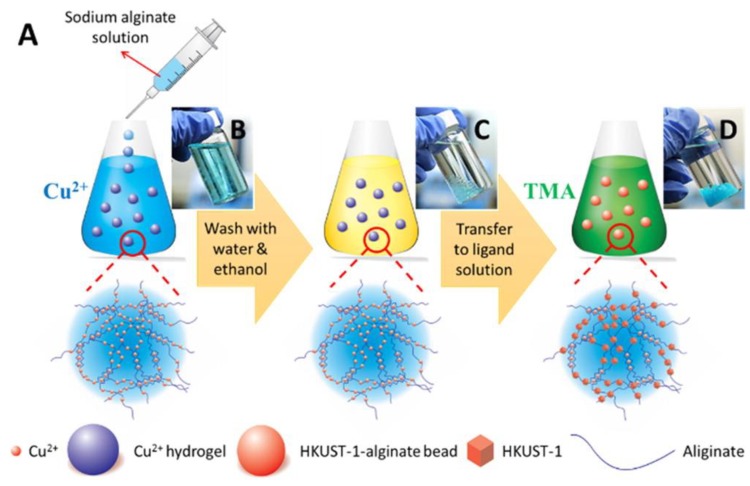
(**A**) Schematic of the preparation of the MOF–alginate composite. Photographs of (**B**) alginate hydrogels cross-linked by Cu^2+^ right after the addition of a sodium alginate aqueous solution to a Cu^2+^ aqueous solution, (**C**) alginate hydrogels cross-linked by Cu^2+^ after being washed with water and ethanol, and (**D**) HKUST-1–alginate hydrogels [159]. Reprinted with permission from Ref. [159]. Copyright American Chemical Society, 2016.

**Figure 9:**
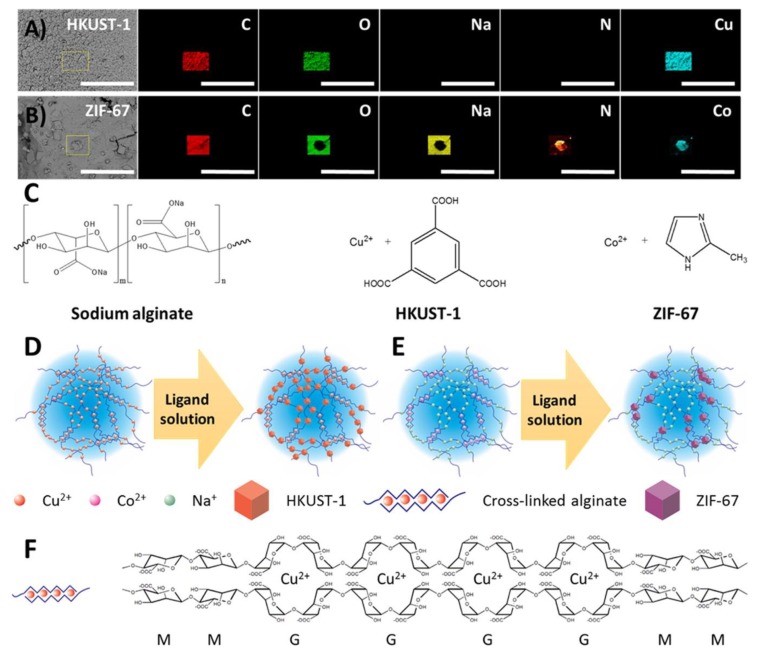
Cross-section backscattered electron images of (**A**) HKUST-1– and (**B**) ZIF-67–alginate composites and their corresponding EDX elemental maps (scale bar of 60 μm). (**C**) Chemical composition of sodium alginate, HKUST-1, and ZIF-67. Schematic of the formation of (**D**) the HKUST-1–alginate composite, (**E**) the ZIF-67–alginate composite, and (**F**) the “egg-box” model of metal ion cross-linked alginate [159]. Reprinted with permission from Ref. [159]. Copyright American Chemical Society, 2016.

Nanofibers of alginates were created by electrospinning in the presence of various synthetic polymers and surfactants [154]. The electrospinning process has been used inexpensively by an efficient technique for the production of nanofibers with the use of biopolymers and other organic substances used in medicine and pharmaceuticals [93,155]. This technique is used to create dressings, drug delivery systems and scaffolds in tissue engineering. The electrospinning creates ultrathin fibers, collected in a random set [156]. The mats that are created during this process are used as wound dressings, catalytic carriers and nanocomposites with many applications [99]. The versatility of the electrospinning technique is so developed that it allows for its use in drug delivery systems and the creation of poly (lactide-co-glycolide) scaffolds, which allows the permanent release of the drug from nanofibres while maintaining their structure and biological activity [86,157]. Nanofibres have also been used in the chemical field as catalysts, sensors and chemical and physical adsorbents. Still, most popular is the use of unique properties of nanofibers prepared using different biopolymers via electrospinning [158]. Several materials have been commercialized under different trademarks such as Coalgan from Brothier Laboratories (France), which is sold as an haemostatic fibers pad for nosebleeds; Algosteril^®^ or Sorban^®^ are also non-woven dressing absorbing rapidly and retains wound fluid resulting in haemostasis and accelerates wound healing. Alginate based hydrogels were also synthesized and used as novel platform for in situ preparation of metal–organic framework [MOF] [159]. In order to establish the feasibility of the synthesis technique, the Hong Kong University of Science and Technology-1 [HKUST-1] –alginate composite was selected as a model system. Figure 8 shows the schematic for the preparation of the MOF–alginate composite and Figure 9 displays the cross-section backscattered electron images. It was concluded from the study that MOF particles can be incorporated into the alginate substrates and for in situ MOF growth, the metal ion cross-linked alginate hydrogels provided outstanding templates. 

### Smart Biopolymers

Intelligent biopolymers are becoming more and more attractive in biotechnology and medicine, as well as in packaging [160,161]. They are used in biomedicine and tissue engineering. Intelligent biopolymers have been reported to play an important role in drug delivery [22,162,163]. They are easy to produce, are a good carrier of nutrients and maintain the stability of the drug [150,164]. It is possible to inject them in vitro as a liquid and they can form a gel at body temperature. Thermosensitive polymers are used to solubilize hydrophobic drugs and are used in the production of preparations with low solubility drugs as a drug carrier. The use of intelligent polymers for drug delivery is promising in view of the mechanism of drug release observed [165]. External and internal stimuli that affect the mechanism include temperature, light irradiation, electric current and intelligent hydrogels that can immobilize enzymes have the ability to gel by phase transition [165]. The chemical signal translates into a mechanical signal causing shrinking or swelling of the gel. This phenomenon is used for the controlled release of the drug. The diffusion of the drug from the beads depends on the condition of the gel [166]. The intelligent polymer is generally integrated with a wall microcapsule or a liposome lipid bilayer. The conformational transition of the polymer affects the integrity of the microcapsule or liposome and allows controlled release of the drug incorporated into the microcapsule or liposome [167]. 

Thanks to the use of drugs released in hydrogels, pharmaceutical aspects can increase productivity, profitability and wide range of applications [70]. One example of an insulin delivery system is a hydrogel comprising an insulin-containing reservoir within a poly (methacrylic acid-g-ethylene glycol) copolymer in which glucose oxidase was immobilized [168,169]. Currently, there is a limited research on fluorinated polymers as a drug carrier in drug delivery applications. Polymer membranes have been used as passive materials for drug release due to their ability to be hydrophilic to hydrophobic in surface wettability. The characteristics of the process are reversible and can go to the initial state [170,171]. The use of lipid based biopolymers for cancer therapy has been recently reviewed [172]. In this article, authors have focused on the advantageous biologic and physicochemical characteristics including controlled drug release, long circulatory half-lives and facile targeted therapy of the natural and synthetic lipid. 

## 4. Summary and Future Prospective

Packaging plays an imperative role in supply chain and has received great attention in number of industries. However, the existing packaging systems are primarily based on synthetic polymers/plastics from fossil resources. Seeing the issues associated with the synthetic polymers/plastics, biomedical and pharmaceutical industries in particular all around the globe are looking for new bio-based sustainable packaging materials that can extend the shelf life of these materials due to the environmental and health issues associated with traditional packaging waste. Increasing the use of sustainable polymeric materials in packaging could address such concerns. The main goals of polymer researchers in the development of new medical and pharmaceutical materials are associated with a reduction in the risk of disease transmission and the spread of infections. The search for new effective antimicrobial dressing materials is constantly growing. Electrospinning is being used in medicine for the production of non-woven structures at the nanoscale. In the electrospinning process, the chemical and physical parameters of the obtained nanofibre mats can be adjusted by dressing the appropriate materials and parameters of the process. The electrospinning process is also influenced by nanoparticles of various natural materials and biopolymers. The addition of natural nanomaterials affects the morphology and size of the electrospun fibre. Along with alginate, the other biopolymers being used in the electrospinning technique include hyaluronic acid, cellulose, silk, gelatin and collagen, to name a few. The mixture of biopolymers and synthetic polymers is also used to create the novel biomaterials with specific properties such as mechanical resistance, thermal stability and barrier resistance. Mixtures with natural polymers may also affect the structural, morphological and subsequent degradation properties of the electrospun fibers

The treatment of disease states with the help of biologically active materials created with the help of electrospun nanofibers is part of modern regenerative medicine. The use of natural materials such as plant oils and extracts means that the nonwovens created in the electrospinning process find many applications with safety of use. It is of great importance that it is possible to optimize process conditions and create nanofibers that meet specific requirements. Among the various materials studied, alginate has very high potential for packaging applications and is being seen as the future of packaging materials. However, a very limited study has been carried out on the use of Alginate based electrospun mats for packing application, and to realize its real potential an extensive study in this direction is needed.
